# Non-alcoholic steatohepatitis and progression of carotid atherosclerosis in patients with type 2 diabetes: a Korean cohort study

**DOI:** 10.1186/s12933-020-01064-x

**Published:** 2020-06-13

**Authors:** Hyeok-Hee Lee, Yongin Cho, Young Ju Choi, Byung Wook Huh, Byung-Wan Lee, Eun Seok Kang, Seok Won Park, Bong-Soo Cha, Eun Jig Lee, Yong-ho Lee, Kap Bum Huh

**Affiliations:** 1 Department of Internal Medicine, Yonsei University College of Medicine, Seoul, Korea; 2 Department of Endocrinology and Metabolism, Inha University School of Medicine, Incheon, Korea; 3 Huh’s Diabetes Center and the 21st Century Diabetes and Vascular Research Institute, Seoul, Korea; 4 Graduate School, Yonsei University College of Medicine, Seoul, Korea; 5 Division of Endocrinology and Metabolism, Department of Internal Medicine, Yonsei University College of Medicine, Seoul, Korea; 6 Department of Systems Biology, Glycosylation Network Research Center, Yonsei University, Seoul, Korea

**Keywords:** Atherosclerosis, Hepatic fibrosis, Metabolic syndrome, Non-alcoholic fatty liver disease, Non-alcoholic steatohepatitis, Type 2 diabetes

## Abstract

**Background:**

There is increasing concern regarding cardiovascular risk in individuals with non-alcoholic fatty liver disease. This study was conducted to evaluate whether hepatic steatosis with or without fibrosis is associated with the progression of carotid atherosclerosis in patients with type 2 diabetes.

**Methods:**

From a longitudinal cohort, we enrolled 1120 patients with type 2 diabetes who underwent repeated carotid artery ultrasonography every 1–2 years. Ultrasonographic findings at baseline and after 6–8 years were compared. Presence of hepatic steatosis was mainly assessed by abdominal ultrasonography; patients with hepatic steatosis were further evaluated for hepatic fibrosis according to fibrosis-4 index. We investigated the association between liver status and atherosclerosis progression.

**Results:**

Of 1120 patients, 636 (56.8%) were classified as having hepatic steatosis at baseline. After 6–8 years, 431 (38.5%) showed atherosclerosis progression. Hepatic steatosis was significantly associated with atherosclerosis progression (adjusted odds ratio[AOR]: 1.370, 95% CI 1.025–1.832; *p *< 0.05). Among patients with hepatic steatosis, only individuals with fibrosis showed significant association with atherosclerosis progression (AOR: 1.615, 95% CI 1.005–2.598; *p *< 0.05). The association between hepatic fibrosis and atherosclerosis progression was significant in all metabolic subgroups regardless of age, body mass index, presence of metabolic syndrome, or insulin sensitivity (all *p* < 0.05). Furthermore, subjects with hepatic steatosis & fibrosis and ≥ 4 components of metabolic syndrome criteria showed markedly increased risk of atherosclerosis progression (AOR: 2.430, 95% CI 1.087–5.458; *p* < 0.05).

**Conclusions:**

Hepatic steatosis with fibrosis is independently associated with the progression of carotid atherosclerosis in patients with type 2 diabetes.

## Background

The prevalence of non-alcoholic fatty liver disease (NAFLD) is rapidly rising relative to increased obesity and/or type 2 diabetes [1]. NAFLD is known to be associated with various complications, such as chronic kidney disease (CKD), cancer, heart failure, or atherosclerosis [2], and cardiovascular complications remain the leading cause of mortality for patients with NAFLD [3–7]. Non-alcoholic steatohepatitis (NASH), one of several categories of NAFLD which is characterized by lobular inflammation and hepatocyte ballooning, produces more significant liver injury like fibrosis or cirrhosis compared to simple NAFLD [8], and patients with NASH were reported to have much higher incidence of coronary artery disease-related mortality [9–11]. In this aspect, there had been numerous previous studies investigating the causal relationship between NAFLD/NASH and carotid atherosclerosis [12–15], regarding carotid atherosclerosis as a surrogate marker of coronary atherosclerosis. Therefore, it might be important to assess hepatic steatosis and fibrosis to identify those at high risk of cardiovascular disease, and to optimally commence medical interventions [16, 17].

This scenario is of special concern in patients with type 2 diabetes, which is known to be associated with higher risk of NAFLD [16, 18]. While NAFLD is an independent risk factor for cardiovascular complications [19], when combined with type 2 diabetes, it further increases the risk of systemic atherosclerosis [3]. Insulin resistance, a characteristic feature of both type 2 diabetes and NAFLD, is known as the key pathophysiology linking type 2 diabetes, NAFLD, and atherosclerosis [2, 20]. However, little is known about the longitudinal effects of NAFLD or NASH on systemic atherosclerosis in type 2 diabetes.

The aim of this study was to investigate the relationship between NAFLD with or without significant fibrosis and the risk of carotid atherosclerosis progression assessed by clinical, laboratory, and repeated imaging findings in type 2 diabetes patients.

## Methods

### Study participants

Participants were recruited from the Seoul Metabolic Syndrome Cohort, of which total 13,296 patients were diagnosed and treated for type 2 diabetes from November 1997 to September 2016 at Huh Diabetes Center as previously described [3, 21]. Patients aged 19 years or older who had undergone repeated carotid artery ultrasonography at 1–2-year intervals for up to 6-8 years were enrolled. Participants were diagnosed with type 2 diabetes according to the American Diabetes Association classification [22]. Patients were excluded for any one of the following criteria: (1) under 19 years of age; (2) diagnosed with type 1 diabetes; (3) pregnant; (4) diagnosed with liver disease other than NAFLD, such as viral or autoimmune hepatitis; and (5) history of heavy alcohol consumption (> 140 g/week). Patients with baseline bilateral carotid artery plaque in whom occurrence of new-onset plaque was difficult to judge in repeat ultrasonography were also excluded. In total, we enrolled 1120 patients with type 2 diabetes who underwent repeat carotid artery ultrasonography at 1–2-year intervals for up to 6–8 years and evaluations for the presence of hepatic steatosis or fibrosis at baseline. All participants provided written informed consent, and the Ethics Committee of the Yonsei University College of Medicine approved this study (4-2019-0270).

### Measurements and definitions of clinical and laboratory parameters

At baseline, we collected information from participants regarding their medical and family history, smoking and alcohol history/consumption, and physical activity level per week. Medication history regarding aspirin, statin, and anti-diabetic drug (insulin, sulfonylurea, metformin, thiazolidinedione) usage was also reviewed. Anthropometrics including weight, height, and waist circumference were obtained by trained nurses who were blinded to patients’ clinical and laboratory data, and blood samples were collected from participants (a) after ≥ 8 h of fasting, and (b) 2 h after a meal. Metabolic parameters including HbA1c, lipid profiles (total cholesterol, high-density lipoprotein cholesterol (HDL-C), low-density lipoprotein cholesterol (LDL-C), triglyceride), blood urea nitrogen (BUN), creatinine, total bilirubin, aspartate/alanine aminotransferase (AST/ALT), total protein, albumin, and platelet count were measured by routine laboratory methods on fresh samples at the same day of collection.

The estimated glomerular filtration rate (eGFR) was derived from the Modification of the Diet in Renal Disease equation (MDRD) [23]. Diagnosis and classification of CKD was based on the Kidney Disease: Improving Global Outcomes (KDIGO) guidelines, and patients with eGFR < 60 mL/min/1.73 m^2^ for > 3 months were diagnosed as CKD stage III–V accordingly [24].

Insulin sensitivity was assessed by calculating the rate constant for plasma glucose disappearance (KITT; %/min) in a short insulin tolerance test [25]. The test was performed at 8:00AM after an overnight fast, and venous blood samples were collected at 0, 3, 6, 9, 12, and 15 min after an intravenous bolus injection of regular insulin (Humulin; Eli Lilly, Indianapolis, IN, USA) at a dosage of 0.1 U/kg. Plasma glucose concentrations were measured immediately after sampling using Beckman glucose analyzer II (Beckman Coulter Inc., Brea, CA, USA), and KITT was determined by calculating the rate of the fall in log-transformed plasma glucose between 3 and 15 min. To prevent potential hypoglycemia, 100 mL of 20% dextrose solution was administered intravenously immediately after testing. Insulin resistance was defined as KITT < 2.5%/min [26].

The diagnosis of metabolic syndrome was made according to a joint interim statement of the International Diabetes Federation Task Force on Epidemiology and Prevention; National Heart, Lung, and Blood Institute; American Heart Association; World Heart Federation; International Atherosclerosis Society; and International Association for the Study of Obesity published in 2009 [27]. Hypertension was defined as systolic blood pressure (BP) ≥ 140 mmHg and/or a diastolic BP ≥ 90 mmHg, or current use of antihypertensive medications. Individuals who drank twice a month or more were defined as regular alcohol consumers, and participants who had ever smoked more than five packs of cigarettes were considered ever-smokers. Regular exercise was defined as moderate to vigorous physical activity for over 30 min more than once a month. Overweight was defined as body mass index (BMI) ≥ 23 kg/m^2^ according to scientific statement from the World Health Organization [28].

### Liver status measurements

Among 1120 participants, 1086 underwent abdominal ultrasonography (iU22; Philips Healthcare, Andover, MA, USA) with a 3.5-MHz transducer after 8 h of fasting. Ultrasound examinations were performed by trained radiologists who were blinded to the patients’ clinical and laboratory information. According to ultrasonographic findings, participants were assessed on whether or not they had hepatic steatosis. The presence of hepatic steatosis in 34 patients who did not undergo abdominal ultrasonography was determined by calculating the Comprehensive Non-Alcoholic Fatty Liver Disease Score (CNS) [29], in which a score ≥ 40 indicated hepatic steatosis. Those with hepatic steatosis were further evaluated for the presence of independent hepatic fibrosis by calculating the fibrosis-4 (FIB-4) index. Significant fibrosis was defined as FIB-4 index ≥ 1.45 in this study [30].

### Carotid atherosclerosis measurements

Every participant underwent repeated carotid ultrasonography every 1–2 years to evaluate carotid atherosclerosis status. We compared the rate of atherosclerosis progression at baseline and at 6–8 years. Both common carotid arteries were examined by high-resolution ultrasonography (LOGIQ7; GE Healthcare, Chicago, IL, USA) by trained technicians who were blinded to the patients’ clinical and laboratory data. The mid and distal common carotid artery was scanned by lateral longitudinal projection, and carotid intima-media thickness (IMT; mm) was measured at three points: far wall of mid; distal common carotid artery; and 1 cm proximal to the carotid bulb. Carotid IMT was defined as the distance between lumen-intima interface and media-adventitia interface, of which the mean value of three measurements on each side was used to represent carotid atherosclerosis status.

Carotid atherosclerosis progression was defined as the appearance of newly developed carotid plaque lesions on repeat ultrasonography. The presence of carotid plaque was defined as meeting any one of the following criteria: (1) carotid IMT of 1.5 mm or higher; (2) protrusion of atherosclerosis into the lumen of artery with ≥ 50% thickness compared to the surrounding area; and (3) presence of distinct area of hyperechogenicity [31].

### Statistical analysis

Baseline characteristics of study participants were analyzed according to liver status: no steatosis; steatosis only; and steatosis with fibrosis. Continuous variables were expressed as mean ± standard deviation (SD) and analyzed with one-way ANOVA for intergroup comparison, followed by Bonferroni test or Dunn procedure for post hoc analysis. All categorical variables were expressed as number (proportion) and compared by Chi square test.

We performed multivariable logistic regression analysis to calculate odds ratio (OR) of carotid atherosclerosis progression according to the presence of hepatic steatosis. After subdividing patients with hepatic steatosis into steatosis only and steatosis with fibrosis, Chi square test was performed to compare the proportion of carotid atherosclerosis progression in each liver status subgroup (no steatosis, steatosis only, and steatosis with fibrosis).

To verify independent association between liver status and carotid atherosclerosis progression, we performed multivariable logistic regression analysis in which various confounding factors were adjusted in a stepwise manner: age and gender were adjusted in Model 2; duration of diabetes, HbA1c, LDL-cholesterol, HDL-cholesterol, statin use, alcohol/smoking consumption, exercise status, systolic BP, diastolic BP, KITT and CKD stage III-V were adjusted in Model 3; and BMI was adjusted in model 4. Models 5 and 6 were built by further adjusting Model 4 with waist circumference and follow-up duration, respectively.

Also, logistic regression analysis was performed to detect the association between liver status and carotid atherosclerosis progression after dividing patients into two subgroups by age (70 years), BMI (overweight status: 23.0 kg/m^2^), presence of metabolic syndrome, or KITT (2.5%/min). Cut-off for age was chosen according to the previous report that cytochrome P450 level declines significantly after age 70 [32], which is known to be very closely related to cholesterol homeostasis and atherosclerosis [33–35]. Finally, study participants were divided into nine subgroups according to liver status and metabolic syndrome criteria, and multivariable logistic regression analysis was performed to calculate OR of carotid atherosclerosis progression in each subgroup. *p* values < 0.05 were considered statistically significant, and all statistical analyses were performed using R version 4.0.0 (R Foundation for Statistical Computing, Vienna, Austria) and IBM SPSS Statistics version 24.0 (IBM Corp., Armonk, NY, USA).

## Results

### Baseline characteristics of study participants

Baseline characteristics are summarized in Table 1. Of 1120 participants, 636 (56.8%) had hepatic steatosis; among them, 222 (19.8%) had significant fibrosis.

**Table 1:** Baseline Characteristics

Study population	No steatosis	Steatosis only	Steatosis with fibrosis	*p* value
N = 1120	N = 484	N = 414	N = 222	
Age, years	55.4 ± 9.4	52.4 ± 9.7^a^	59.8 ± 7.8^ab^	< 0.001
Male, n (%)	216 (44.6%)	211 (51.0%)	104 (46.8%)	0.163
Weight, kg	61.1 ± 9.4	68.8 ± 11.8^a^	68.8 ± 10.7^a^	< 0.001
Height, cm	162.1 ± 8.6	163.6 ± 8.9^a^	162.4 ± 8.5	0.033
BMI, kg/m^2^	23.2 ± 2.8	25.6 ± 3.0^a^	26.1 ± 3.2^a^	< 0.001
Waist Circumference, cm	79.2 ± 7.5	85.8 ± 8.0^a^	87.2 ± 7.8^a^	< 0.001
Metabolic Syndrome, n/total n (%)	174/466 (37.3%)	282/404 (69.8%)	150/221 (67.9%)	< 0.001
Regular alcohol consumption, n/total n (%)	182/437 (41.6%)	183/392 (46.7%)	70/204 (34.3%)	0.014
Smoking, ever, n/total n (%)	166/420 (39.5%)	174/384 (45.3%)	70/194 (36.1%)	0.072
Regular exercise, n/total n (%)	135/383 (35.2%)	172/360 (47.8%)	78/187 (41.7%)	0.002
Hypertension, n (%)	130 (26.9%)	135 (32.6%)	97 (43.7%)	< 0.001
SBP, mmHg	131.1 ± 16.7	135.0 ± 16.9^a^	138.3 ± 16.4^a^	< 0.001
DBP, mmHg	84.3 ± 10.6	88.3 ± 11.2^a^	87.4 ± 10.6^a^	< 0.001
Duration of diabetes, years	7.2 ± 6.9	5.5 ± 5.3^a^	6.1 ± 5.5	< 0.001
HbA1c, %	8.3 ± 2.1	8.7 ± 1.9^a^	8.0 ± 1.6^b^	< 0.001
HbA1c, mmol/mol	67.0 ± 23.0	72.0 ± 20.8^a^	64.0 ± 17.5^b^	< 0.001
KITT, %/min	2.4 ± 1.0	1.9 ± 0.8^a^	1.8 ± 0.7^a^	< 0.001
Total Cholesterol, mg/dL	188.6 ± 38.5	201.8 ± 45.1^a^	197.7 ± 39.3^a^	< 0.001
Triglyceride, mg/dL	115.7 ± 63.3	172.3 ± 140.0^a^	157.7 ± 89.2^a^	< 0.001
HDL-C, mg/dL	54.2 ± 14.8	48.4 ± 12.2^a^	50.6 ± 12.8^a^	< 0.001
LDL-C, mg/dL	110.1 ± 32.3	118.9 ± 35.7^a^	113.6 ± 35.3	0.002
BUN, mg/dL	17.5 ± 7.3	16.9 ± 5.0	18.7 ± 8.2^b^	0.005
Creatinine, mg/dL	0.8 ± 0.2	0.8 ± 0.3	0.8 ± 0.2^a^	0.007
eGFR (MDRD), mL/min/1.73 m^2^	94.5 ± 30.9	93.0 ± 28.0	88.0 ± 27.4^a^	0.022
Total Bilirubin, mg/dL	0.9 ± 0.3	0.8 ± 0.3	1.0 ± 0.5^ab^	0.002
AST, IU/L	25.0 ± 12.6	24.4 ± 8.0	36.7 ± 17.6^ab^	< 0.001
ALT, IU/L	23.6 ± 15.9	30.5 ± 14.9^a^	37.0 ± 25.2^ab^	< 0.001
Total Protein, mg/dL	7.3 ± 0.4	7.3 ± 0.4	7.4 ± 0.5^ab^	0.006
Albumin, mg/dL	4.3 ± 0.4	4.4 ± 0.3	4.4 ± 0.3	0.068
Platelet,/uL	210.7 ± 59.2	240.0 ± 53.4^a^	183.3 ± 38.6^ab^	< 0.001
Insulin use, n (%)	47 (9.7%)	24 (5.8%)	15 (6.8%)	0.076
SU use, n (%)	240 (49.6%)	191 (46.1%)	140 (63.1%)	< 0.001
Metformin use, n (%)	172 (35.5%)	156 (37.7%)	93 (41.9%)	0.270
TZD use, n (%)	57 (11.8%)	25 (6.0%)	21 (9.5%)	0.012
Statin use, n (%)	63 (13.0%)	53 (12.8%)	25 (11.3%)	0.797
Aspirin use, n (%)	62 (12.8%)	58 (14.0%)	35 (15.8%)	0.568
Carotid IMT, mm	0.75 ± 0.15	0.76 ± 0.15	0.81 ± 0.14^ab^	< 0.001
Presence of plaque, n (%)	153 (31.6%)	127 (30.7%)	80 (36.0%)	0.365

Variables are shown as mean ± SD or n (%). ALT, alanine aminotransferase; AST, aspartate aminotransferase; BMI, body mass index; BUN, blood urea nitrogen; DBP, diastolic blood pressure; eGFR, estimated glomerular filtration rate; HDL-C, high-density lipoprotein cholesterol; IMT, intima-media thickness; KITT, rate constant for plasma glucose disappearance; LDL-C, low-density lipoprotein cholesterol; MDRD, modification of diet in renal disease equation; SBP, systolic blood pressure; SD, standard deviation; SU, sulfonylurea; TZD, thiazolidinedione

^a^*p* values < 0.05 versus no steatosis

^b^*p* values < 0.05 versus steatosis only

The mean age of subjects with hepatic steatosis and fibrosis was 59.8 (± 7.8) years, which was significantly higher compared to the other subgroups (*p* < 0.001). BMI (kg/m^2^) was higher in those with both hepatic steatosis and fibrosis (26.1 ± 3.2) or only steatosis (25.6 ± 3.0) compared to those without steatosis (23.2 ± 2.8) (*p* < 0.001). 282 (69.8%) participants with only hepatic steatosis and 150 (67.9%) participants with both hepatic steatosis and fibrosis had metabolic syndrome, while only 174 (37.3%) participants had metabolic syndrome among those without hepatic steatosis (*p* < 0.001). KITT (%/min) was 2.4 (± 1.0) in subjects with no hepatic steatosis, which was significantly higher compared to the subgroup with only steatosis (1.9 ± 0.8) or with both steatosis and fibrosis (1.8 ± 0.7) (*p* < 0.001), indicating that participants without hepatic steatosis were more insulin-sensitive than those in other two subgroups.

Participants with both steatosis and fibrosis showed lower eGFR (88.0 ± 27.4) compared to those with no steatosis (94.5 ± 30.9) (*p* = 0.018), but it was not significantly lower than the steatosis only group (93.0 ± 28.0) (*p* = 0.122). There was no significant difference by sex or statin use between the three subgroups.

The mean carotid IMTs (mm) at baseline were 0.75 ± 0.15, 0.76 ± 0.15, and 0.81 ± 0.14 in patients with no hepatic steatosis, steatosis only, and steatosis with fibrosis, respectively (*p* < 0.001). The proportion of participants with carotid plaque at baseline was not significantly different between the three subgroups (*p* = 0.365).

### Association between hepatic steatosis and progression of carotid atherosclerosis

**Fig. 1:**
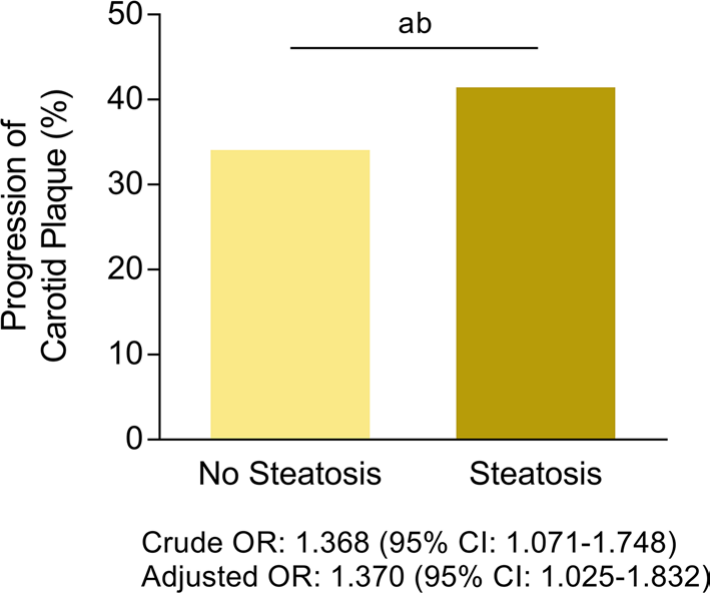
Progression of Carotid Atherosclerosis by Presence of Hepatic Steatosis. Odds ratio of carotid atherosclerosis progression according to the presence of hepatic steatosis. The result is adjusted for age, gender, systolic blood pressure, diastolic blood pressure, duration of diabetes, HbA1c, rate constant for plasma glucose disappearance (KITT), chronic kidney disease stage III–V, total cholesterol, statin use, and alcohol history. Levels of significance: ^a^*p* = 0.012 (crude); ^b^*p* = 0.034 (adjusted) (Logistic regression analysis)

The presence of hepatic steatosis increased the risk of carotid plaque progression (OR: 1.368, 95% CI 1.071–1.748; *p* = 0.012). This result persisted after adjusting for age, gender, systolic BP, diastolic BP, duration of diabetes, HbA1c, KITT, CKD stage III-V, total cholesterol, statin use, and alcohol history (adjusted odds ratio[AOR]: 1.370, 95% CI 1.025–1.832; *p* = 0.034) (Fig. 1).

### Presence of hepatic fibrosis and carotid atherosclerosis progression in patients with NAFLD

**Fig. 2:**
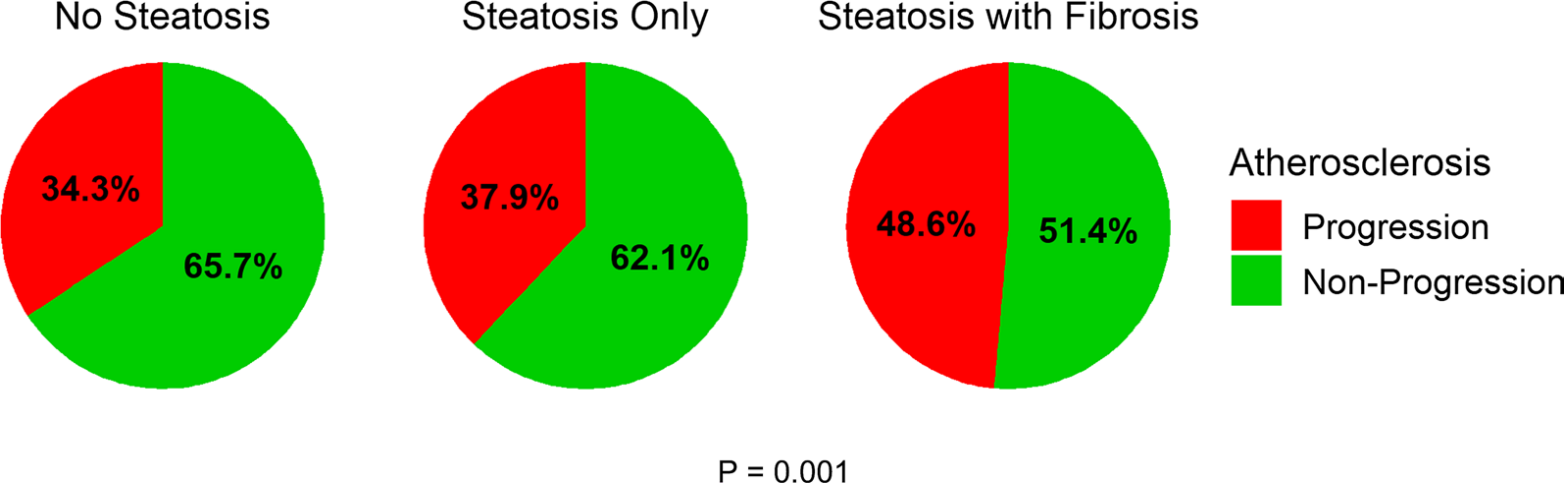
Progression of Carotid Atherosclerosis by Presence of Hepatic Steatosis and Fibrosis. Proportion of carotid atherosclerosis progression in patients with no steatosis, steatosis only, and steatosis with fibrosis. Levels of significance: *p* = 0.001 (Chi square test)

The number (%) of patients with carotid plaque progression after 6–8 years was 166 (34.3%), 157 (37.9%), and 108 (48.6%), respectively, among those with no hepatic steatosis, steatosis only, and steatosis with fibrosis. The difference was statistically significant (*p* = 0.001) (Fig. 2).

**Fig. 3:**
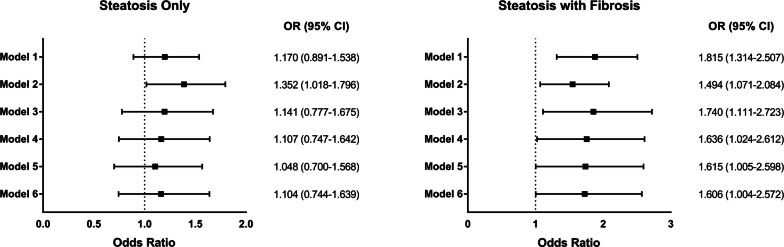
Risk of Carotid Atherosclerosis Progression According to Hepatic Status. Odds ratios of carotid atherosclerosis progression according to hepatic status. Model 1 = Crude model without any adjustment; Model 2 = Model 1 + age, gender; Model 3 = Model 2 + duration of diabetes, HbA1c, low-density lipoprotein cholesterol, high-density lipoprotein cholesterol, statin use, alcohol/smoking consumption, exercise status, systolic blood pressure, diastolic blood pressure, rate constant for plasma glucose disappearance (KITT), chronic kidney disease stage III-V; Model 4 = Model 3 + body mass index; Model 5 = Model 4 + waist circumference; Model 6 = Model 4 + follow-up duration. (Logistic regression analysis)

To further investigate whether presence of hepatic fibrosis is independently associated with the progression of carotid plaque in patients with NAFLD, we performed multivariable logistic regression analyses in a stepwise manner. With no adjustment (Model 1), hepatic steatosis with fibrosis was statistically significantly associated with carotid plaque progression (OR: 1.815, 95% CI 1.314–2.507; *p* < 0.001), whereas steatosis only was not significant (OR: 1.170, 95% CI 0.891–1.538; *p* = 0.259). Steatosis with fibrosis was still significantly associated with carotid plaque progression after adjusting for age, gender (Model 2. AOR: 1.494, 95% CI 1.071–2.084; *p* = 0.018), duration of diabetes, HbA1c, LDL-cholesterol, HDL-cholesterol, statin use, alcohol/smoking consumption, exercise status, systolic BP, diastolic BP, KITT, CKD stage III-V (Model 3. AOR: 1.740, 95% CI: 1.111-2.723; *p* = 0.015), BMI (Model 4. AOR: 1.636, 95% CI 1.024–2.612; *p* = 0.039), and waist circumference (Model 5. AOR: 1.615, 95% CI 1.005–2.598; *p* = 0.048). Further adjusting Model 4 for follow-up duration still did not alter statistical significance of the result (Model 6. AOR: 1.606, 95% CI 1.004–2.572; *p* = 0.048) (Fig. 3).

### Risk of carotid atherosclerosis progression according to metabolic profiles

**Fig. 4:**
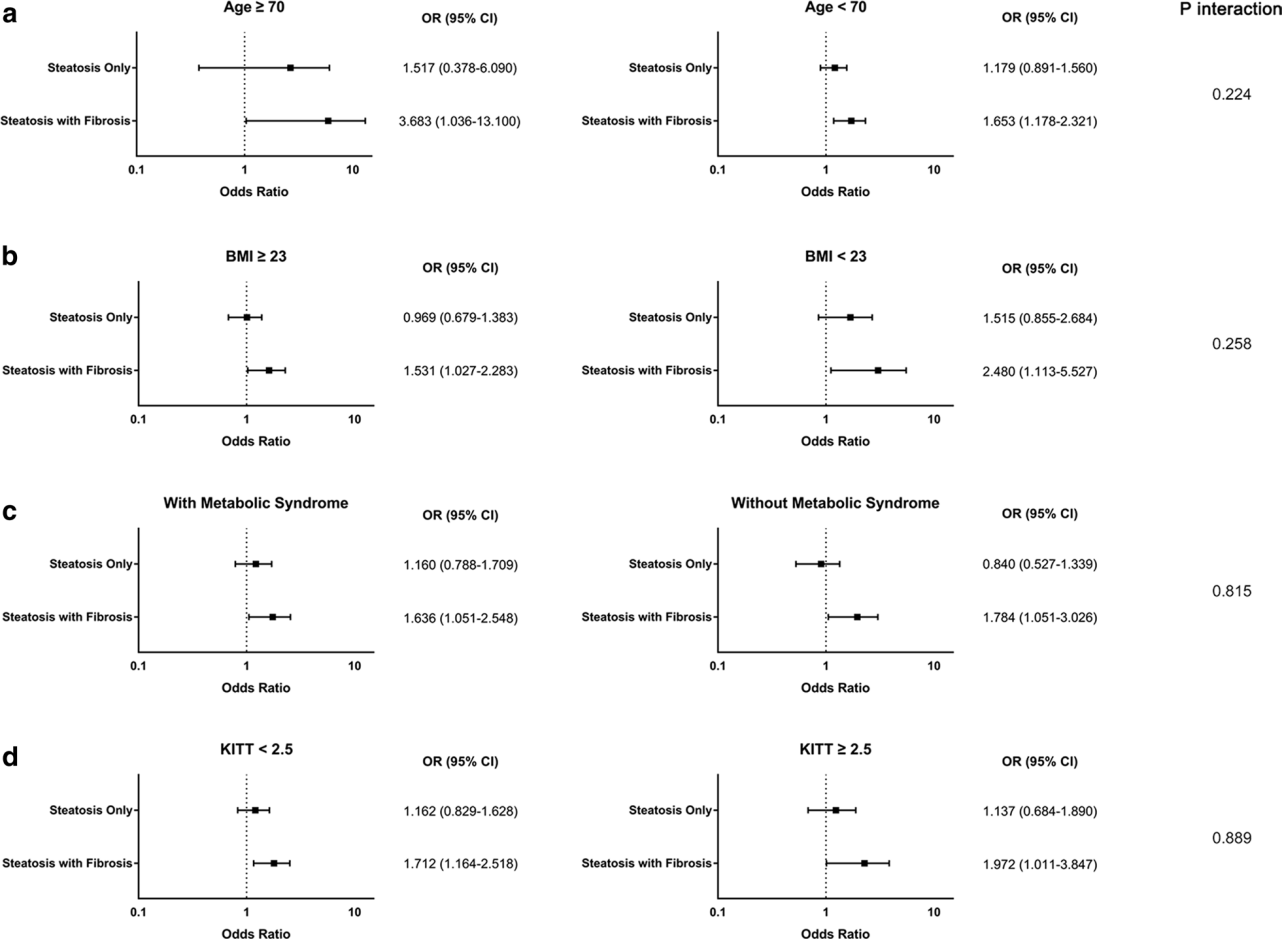
Hepatic Status and Carotid Atherosclerosis Progression by Metabolic Confounders. Odds ratios of carotid atherosclerosis progression according to hepatic status and **a** Age. Level of significance: *p* interaction = 0.224. **b** Body mass index. Level of significance: *p* interaction = 0.258. **c** Presence of metabolic syndrome. Level of significance: *p* interaction = 0.815. **d** Insulin sensitivity. Level of significance: *p* interaction = 0.889. Risk estimates were calculated in each subgroup using patients with no steatosis as a reference (Logistic regression analysis). BMI, body mass index; KITT, rate constant for plasma glucose disappearance

To examine the presence of potential effect modification, we analyzed the risk of carotid atherosclerosis progression according to several metabolic factors. Overall, the analysis showed no difference between metabolic subgroups, whether they were divided by age, BMI, presence of metabolic syndrome, or insulin resistance (all *p* interaction > 0.05). In detail, hepatic steatosis without fibrosis was not associated with the progression of carotid atherosclerosis in any metabolic subgroup. However, patients with combined hepatic steatosis & fibrosis showed statistically significantly higher risk of carotid atherosclerosis progression regardless of age (OR: 3.683, 95% CI 1.036–13.100 in subgroup with age ≥ 70; OR: 1.653, 95% CI 1.178–2.321 in subgroup with age < 70), BMI (OR: 1.531, 95% CI 1.027–2.283 in subgroup with BMI ≥ 23; OR: 2.480, 95% CI 1.113–5.527 in subgroup with BMI < 23), presence of metabolic syndrome (OR: 1.636, 95% CI 1.051–2.548 in subgroup with metabolic syndrome; OR: 1.784, 95% CI 1.051–3.026 in subgroup without metabolic syndrome), or insulin sensitivity (OR: 1.712, 95% CI 1.164–2.518 in subgroup with KITT < 2.5 (insulin resistant); OR: 1.972, 95% CI: 1.011-3.847 in subgroup with KITT ≥ 2.5 (insulin sensitive)). There was no effect modification by metabolic factors (*p* interaction = 0.224, 0.258, 0.815, and 0.889 for age, BMI, presence of metabolic syndrome, and insulin sensitivity, respectively) (Fig. 4).

**Fig. 5:**
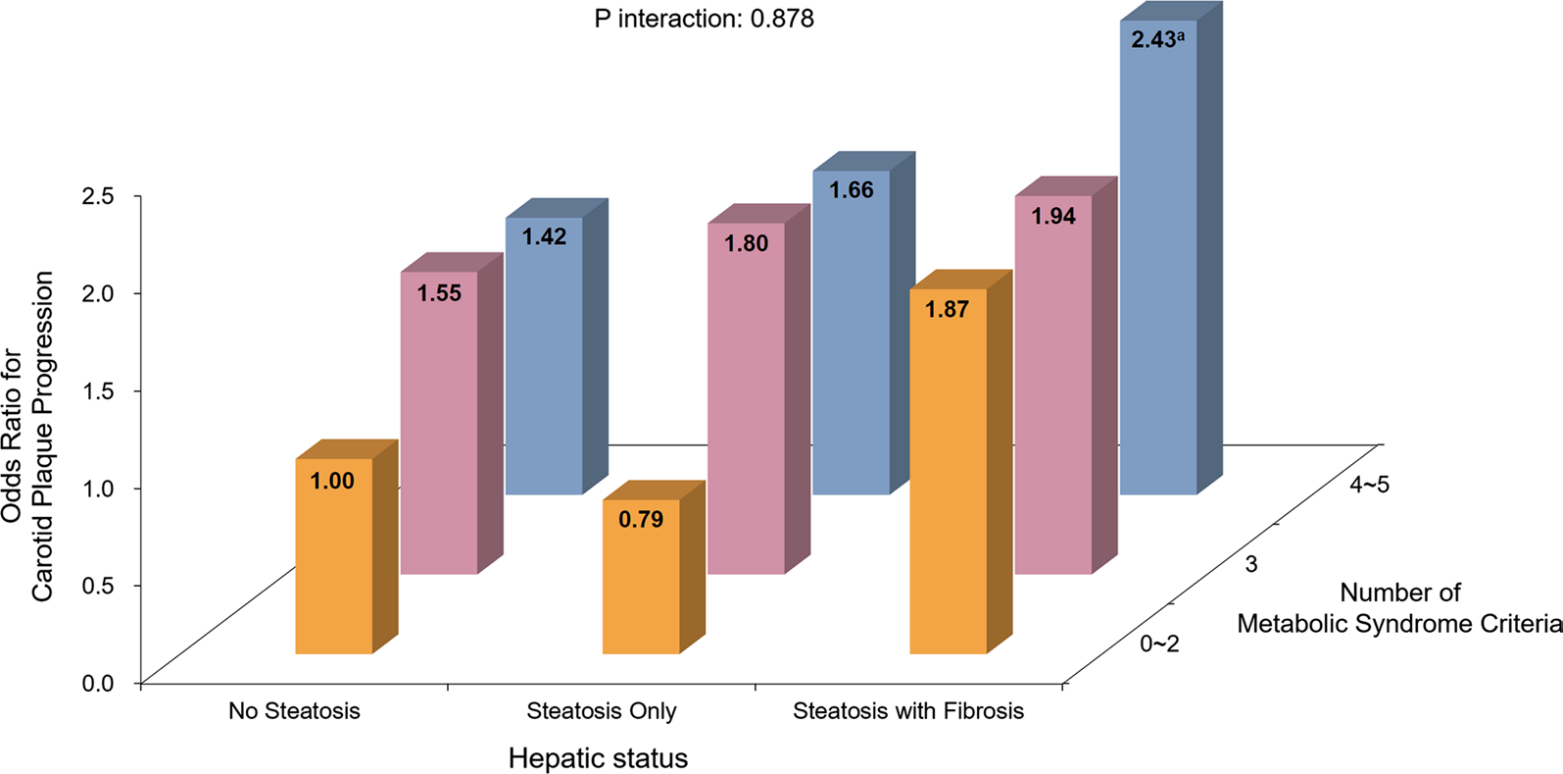
Combinatorial Effect of Liver Status and Metabolic Syndrome Criteria on Carotid Atherosclerosis Progression. Odds ratios of carotid atherosclerosis progression according to hepatic status and metabolic syndrome criteria. Odds ratio was calculated in each subgroup using the subgroup with 0–2 metabolic syndrome criteria and no hepatic steatosis as a reference. The result is adjusted for age, gender, duration of diabetes, HbA1c, low-density lipoprotein cholesterol, high-density lipoprotein cholesterol, statin use, alcohol/smoking consumption, exercise status, systolic blood pressure, diastolic blood pressure, rate constant for plasma glucose disappearance (KITT), chronic kidney disease stage III–V, body mass index, waist circumference, and follow-up duration. Levels of significance: ^a^*p* = 0.031, *p* interaction = 0.878 (Logistic regression analysis)

Consecutively, to investigate the combinatorial effects of cardiometabolic risk factors and liver status, participants were divided into 9 subgroups according to liver status (no steatosis, steatosis only, steatosis with fibrosis) and the number of metabolic syndrome criteria met (0–2, 3, 4–5). In each metabolic syndrome criteria subgroup, the risk of carotid atherosclerosis progression was generally higher in subjects with hepatic steatosis, and far higher in those with both hepatic steatosis and fibrosis. Similarly, in each liver status subgroup, a higher number of metabolic syndrome criteria generally correlated with higher risk of carotid atherosclerosis progression. Compared to those with 0–2 metabolic syndrome criteria and no hepatic steatosis, subjects with 4–5 metabolic syndrome criteria and both steatosis and fibrosis were at significantly higher risk of carotid atherosclerosis progression (AOR: 2.430, 95% CI 1.087–5.458, *p* = 0.031) (Fig. 5).

## Discussion

### Principal findings and clinical implications

Ultrasonography is now widely accepted as a useful screening tool to detect carotid artery plaque and predict cardiovascular events [31, 36]. With serial carotid ultrasonography of patients with type 2 diabetes at 1–2-year intervals for up to 6–8 years, this study demonstrated that hepatic steatosis with significant fibrosis was strongly associated with the progression of carotid artery atherosclerosis, even in relatively metabolically-healthy patients. Our results have also shown that hepatic fibrosis and metabolic syndrome accelerate atherosclerosis progression independently of each other, delivering an additive effect when combined together.

Based on these findings, we suggest that hepatic fibrosis can be an independent risk factor for atherogenesis acceleration, and its identification by clinical indicators may be helpful to predict the risk of atherosclerosis progression. Also, patients who are already diagnosed with metabolic syndrome should especially be aware of hepatic fibrosis to blunt profoundly higher risk of atherosclerosis produced by the combined effect of hepatic fibrosis and metabolic syndrome.

### Results in relation to other studies

NAFLD is considered a ‘hepatic manifestation of metabolic syndrome’. It is very closely related to type 2 diabetes or metabolic syndrome, and the main pathophysiology underlying this relationship is known to be insulin resistance [37, 38]. The association is considerably strong that NAFLD is present even in obese adolescents with dysglycemia [39]. NAFLD and metabolic syndrome can be considered to have similar effects on arteries, which accelerate atherogenesis via inflammation [40, 41], increased oxidative stress [42], atherogenic dyslipidemia [43], imbalance of adipokines [44], and hypercoagulable status [45]. As a result, NAFLD patients present lower reactive hyperemia index and higher pulse wave velocity [46], and NAFLD can serve as a strong predictor of coronary artery calcification in metabolically healthy subjects [47].

In consequence, NAFLD was reported as an independent risk factor for cardiovascular disease in the general population [7, 48]. After several efforts to prove this relationship via carotid ultrasonography, it was discovered that NAFLD was significantly associated with carotid stenosis in Chinese population [49], increased carotid IMT in type 2 diabetes patients with insulin resistance [3], and higher prevalence of carotid plaque [14]. Moreover, NAFLD was shown to be associated with higher cardiovascular risk in terms of carotid IMT and dyslipidemia even in nondiabetic patients [50]. In the present study, we focused on the long-term effect of NAFLD with or without significant fibrosis on atherosclerosis by repeated carotid ultrasonography. The results showed that progression of carotid atherosclerosis after 6-8 years occurred more frequently in patients with NASH. Although it has been shown that visceral obesity is associated with cardiometabolic comorbidities of type 2 diabetes, NAFLD, or atherosclerotic cardiovascular diseases [51], further adjustment for waist circumference along with other common metabolic factors did not alter the significance of association between hepatic fibrosis and atherosclerosis progression in our analysis.

To our knowledge, this is the first report to demonstrate that hepatic fibrosis is significantly associated with the progression of carotid artery atherosclerosis in subjects with type 2 diabetes. It indicates that not only presence—but also severity—of metabolic liver disease can affect the risk of cardiovascular complications. Previous long-term studies showed that risk of coronary artery disease-related mortality was much higher in patients with NASH (12–16%) [9, 52] compared to NAFLD (1–3%) [10, 53], and these findings were consistent with a recent meta-analysis in which increased NAFLD severity produced higher risk of cardiovascular complications [11], or recent large Korean population-based cohort study which demonstrated the linear association between fatty liver index (FLI) and major adverse cardiovascular events [54]. Altered lipidomics and increased hepatic production of prothrombogenic factors, including fetuin-A in patients with fibrosing NASH, can be potential contributors to the link between NASH and cardiovascular diseases [55].

In addition, the association between hepatic fibrosis and risk of atherosclerosis progression was significant in all metabolic subgroups regardless of age, BMI, presence of metabolic syndrome, or insulin sensitivity. It indicates that hepatic fibrosis may serve as a predictive marker for increased susceptibility to atherosclerosis progression even with less evidence of systemic metabolic alterations, implicating the possible presence of systemic profibrogenic stimuli that accelerate atherogenesis in patients with hepatic fibrosis [56].

Conversely, there was no incremental risk of atherosclerosis progression in hepatic steatosis without fibrosis in type 2 diabetes patients. This finding was similar to that of a previous study in which patients with hepatic steatosis and no additional feature of liver injury were found to follow a relatively benign clinical course, with mortality similar to the general population [57]. Although steatosis without fibrosis was not associated with increased risk of atherosclerosis progression in this study, repeat ultrasonography was not performed beyond 8 years, making it difficult to predict longer-term effect of steatosis without fibrosis on the risk of atherosclerosis progression. Since high rates of fibrosis progression have been demonstrated in patients with steatosis [58], it would be important to consider its clinical significance and to manage it appropriately without overlooking the risk of cardiovascular complication.

### Strengths & limits

This study has several distinguishing strengths. First, we analyzed long-term results of carotid ultrasonography in subjects with type 2 diabetes. Most previous studies using carotid ultrasonography were cross-sectional and insufficient to determine a causal relationship. In addition, since this study was a hospital-based cohort study conducted at a single institution, the participants were managed and evaluated under standardized conditions and practices.

A major limitation of this study is the fact that a biochemical scoring system, rather than liver biopsy, was used to evaluate hepatic fibrosis, which is currently not a gold-standard for the diagnosis. However, FIB-4 index was initially validated by comparing the results to that of liver biopsy [30], and they were shown to have fairly high accuracy to predict hepatic fibrosis [59, 60]. Secondly, this study analyzed the findings of carotid deterioration using ultrasonography, one of the major surrogate markers of cardiovascular disease. However, our methods did not allow for the investigation of cardiovascular events that could represent a direct outcome of atherosclerosis progression. Also, antiplatelet, antihyperglycemic agent, or fibric acid usage were not adjusted in our models, which are potential confounding factors that could influence our results. In addition, we did not consider how participants’ metabolic factors changed over time in our analyses. Finally, this study was based on a single-center cohort of Koreans with a relatively small number of participants. Therefore, further larger studies including other ethnic populations are needed to validate the results, as well as to confirm generalizability of the results.

## Conclusions

In conclusion, hepatic steatosis with significant fibrosis was independently associated with the progression of carotid atherosclerosis in patients with type 2 diabetes. The association was still significant in subgroups of patients who were metabolically healthy, and it became more prominent relative to criteria for metabolic syndrome. Identification of hepatic steatosis with significant fibrosis may be helpful to predict and prevent the risk of atherosclerosis progression in individuals with type 2 diabetes.

## Data Availability

The datasets generated and/or analyzed during the current study are available from the corresponding author upon reasonable request.

## Abbreviations

ALT: Alanine aminotransferase
AST: Aspartate aminotransferase
BMI: Body mass index
BP: Blood pressure
BUN: Blood urea nitrogen
CKD: Chronic kidney disease
CNS: Comprehensive non-alcoholic fatty liver disease score
eGFR: Estimated glomerular filtration rate
FIB-4: Fibrosis-4
FLI: Fatty liver index
HDL-C: High-density lipoprotein cholesterol
IMT: Intima-media thickness
KITT: Rate constant for plasma glucose disappearance
LDL-C: Low-density lipoprotein cholesterol
MDRD: Modification of the Diet in Renal Disease
NAFLD: Non-alcoholic fatty liver disease
NASH: Non-alcoholic steatohepatitis
